# Treatment outcomes of spontaneous rupture of hepatocellular carcinoma with hemorrhagic shock: a multicenter study

**DOI:** 10.1186/s40064-016-2762-8

**Published:** 2016-07-16

**Authors:** Feng Zhong, Xin-Sheng Cheng, Kun He, Shi-Bo Sun, Jie Zhou, Hai-Ming Chen

**Affiliations:** Department of Hepatobiliary Surgery, Nanfang Hospital, Southern Medical University, No. 1838, North Guangzhou Avenue, Guangzhou, 510515 Guangdong Province China; Department of Hepatobiliary Surgery, Shenzhen Hospital of Southern Medical University, Shenzhen, 518100 Guangdong Province China; Department of General Surgery, Jiangmen People’s Hospital, Jiangmen, 529000 Guangdong Province China

**Keywords:** Hepatocellular carcinoma, Rupture, Hemorrhagic shock, Conservative treatment, Hepatectomy, Transcatheter arterial embolization

## Abstract

**Background:**

Spontaneous rupture is one of the most fatal complications of HCC. The incidence of HCC still remains a significant health problem in Eastern Asia. Many studies have shown that the in-hospital or 30-day mortality rates are as high as 25–100 %. It is often difficult to stratify these patients based on clinical manifestations and biochemical data, for deciding on an appropriate treatment strategy, especially when the patient’s hemodynamic status is unstable. This study aimed to explore the clinical outcomes of treatment of spontaneously ruptured hepatocellular carcinoma with hemorrhagic shock.

**Methods:**

One hundred and sixty two patients with hemorrhagic shock secondary to spontaneous rupture of hepatocellular carcinoma were included in this retrospective study. The therapeutic methods included conservative treatment, transcatheter arterial embolization (TAE) and hepatectomy. The outcomes in terms of 30 day and 1 year survival were analyzed.

**Results:**

Thirty five (21.6 %) received only conservative management, TAE was performed in 48 (29.6 %) and partial hepatectomy (emergency and staged) in 106 (65.4 %) patients. The 30-day survival rate was lower in patients receiving conservative treatment (8.6 %) than in those receiving either hepatectomy or TAE (88.2 %; *P* < 0.001). Conservative treatment was associated with poorer long-term survival (0 % at 1 year) when compared to those receiving either hepatectomy or TAE (54.3 % at 1 year; *P* < 0.001). The survival rates at 30 days and 1 year were 92.5 % and 59.4 % for the patients who underwent hepatectomy, which were significantly higher (66.7 and 28.6 % respectively) than those receiving TAE alone (*P* = 0.003 and *P* = 0.009, respectively). Multivariate Cox-regression analysis showed that hepatectomy and TAE were significant protective factors for survival as compared with conservative treatment (all *P* < 0.01).

**Conclusions:**

Partial hepatectomy, tended to provide better survival than transcatheter arterial embolization alone or conservative treatment in the management of patients with hemorrhagic shock secondary to spontaneous rupture of hepatocellular carcinoma.

## Background

Spontaneous rupture is one of the most fatal complications of hepatocellular carcinoma (HCC) and possesses a geographically variable incidence. In Western countries, the incidence of ruptured HCC is less than 3 % (Yamagata et al. 1995; Vergara et al. 2000; Clarkston et al. 1998). However, in Asia, spontaneous rupture occurs in 2.3–26 % of all HCC case (Chen et al. 1995; Miyamoto et al. 1991; Dewar et al. 1991; Yuan et al. 2004; Liu et al. 2001). The incidence of ruptured HCC has reduced in the recent past, owing to the early diagnosis, but this condition still remains a significant health problem in Eastern Asia. Many studies have shown that the in-hospital or 30-day mortality rates are as high as 25–100 % (Liu et al. 2001; Chen et al. 2005; Darnis et al. 2014; Kirikoshi et al. 2009; Lai and Lau 2006; Lee et al. 2014; Lin et al. 2014; Tan et al. 2006). In most cases, spontaneous rupture of HCC occurs suddenly, and these patients usually present to the emergency department with abdominal pain and/or hypotension (Chen et al. 2005; Darnis et al. 2014; Lai and Lau 2006; Lin et al. 2014; Hsueh et al. 2012; Yang et al. 2013). It is often difficult to stratify these patients based on clinical manifestations and biochemical data, for deciding on an appropriate treatment strategy, especially when the patient’s hemodynamic status is unstable. Studies have shown that several therapies such as hepatectomy, surgical ligation of the hepatic artery, perihepatic packing, and transcatheter arterial embolization (TAE) improve clinical outcomes (Vergara et al. 2000; Kirikoshi et al. 2009; Lai and Lau 2006; Choi et al. 2001). The tumor characteristics and liver function guide the choice of treatment and affect prognosis (Vergara et al. 2000; Chen et al. 2005; Lai and Lau 2006; Choi et al. 2001). Some experts prefer TAE or conservative management (Tan et al. 2006; Yang et al. 2013; Kim et al. 2012; Kung et al. 2008; Li et al. 2009; Yeh et al. 2002), while others consider emergency hepatectomy or staged hepatectomy after TAE for ruptured HCC to be a better therapeutic option (Lee et al. 2014; Lin et al. 2014; Hsueh et al. 2012; Yang et al. 2013; Zhang et al. 2015; Li et al. 2007; Jin et al. 2013). To the best of our knowledge, till date, no prospective randomized controlled trials to directly compare these method of hemostasis have been published. Additionally, there is no consensus on the most appropriate treatment modality.

There are a few multicenter studies on spontaneously ruptured HCC, but none investigated the treatment outcomes for this condition with associated hemorrhagic shock in a large study population in a multicenter setting. In this study, we analyzed the data of spontaneously ruptured HCC presenting with hemorrhagic shock during a 10-year period at three medical centers. This study aimed to explore the immediate and long term clinical outcomes of emergency hepatectomy, staged hepatectomy, TAE alone and conservative treatment in spontaneously ruptured hepatocellular carcinoma with hemorrhagic shock.

## Methods

A total of 162 patients with a diagnosis of spontaneous rupture of HCC with hemorrhagic shock admitted to Nanfang Hospital (42 patients; a university hospital), Nanshan Hospital (89 patients; a university hospital) and Jiangmen People’s Hospital (31 patients; a central hospital), China between January 2004 and January 2014, were enrolled. After approval from the Nanfang Hospital Institutional Review Board, the Nanshan Hospital Ethical Committee and the Jiangmen People’s Hospital Ethical Committee, the data was extracted from database records and hospital charts. One hundred and six patients were diagnosed with ruptured HCC based on intraoperative exploration findings. In the rest of the patients (56), diagnosis was made on the basis of dynamic contrast-enhanced abdominal CT with typical findings of a ruptured HCC, including active extravasation of contrast material, HCC with a surrounding perihepatic hematoma showing high attenuation, protrusion of the contour, focal discontinuity of the hepatic surface, and an enucleation sign or identification of hemorrhagic fluid by abdominal paracentesis (with more than 100,000,000 erythrocytes/mL of ascetic fluid). Circulatory shock was defined as a systolic blood pressure <90 mmHg (1 mmHg = 0.133 kPa) and a pulse rate >100 beats/min.

The clinical data was retrospectively reviewed from the medical records. The patients were diagnosed based on medical history, physical examination, contrast-enhanced abdominal CT scan, and abdominal sonography with paracentesis. The demographics, past medical history, hemodynamic status, associated laboratory data, diagnostic methods, Modified Union for International Cancer Control (UICC) stage (Liver Cancer Study Group of Japan 1989), tumor characteristics on imaging, and treatment modalities were recorded.

### Evaluation of patients

The patients were monitored closely either in the ward or the intensive care unit. After initial fluid resuscitation, all patients were classified either as hemodynamically stable or unstable. Liver function was monitored closely before definite treatment decision making. Tumor characteristics, including greatest dimension, location of rupture, and number of intrahepatic nodules, were evaluated by ultrasonography or abdominal CT scan.

### Treatment modality of ruptured HCCs

The patients and their family members were explained about the four management options of emergency hepatectomy, staged hepatectomy, TAE and best supportive care (conservative management) with relative benefits and risks of each. Criteria for emergency surgical treatment included: (1) patients with stable hemodynamic after initial fluid resuscitation; (2) patients were expectedly able to tolerate surgery; (3) patients and their relatives agreed to undergo surgery. Criteria for TAE treatment were: (1) patients were expectedly able to tolerate TAE treatment; (2) patients and their relatives agreed to undergo TAE treatment. Criteria for staged surgery included: (1) patients with stable hemodynamic after TAE therapy; (2) patients can tolerate surgery; (3) the tumor was expectedly to be removed, (4) patients and their relatives agreed to undergo surgery. Criteria for conservative treatment were: (1) patients and their relatives did not agree to undergo either surgery or TAE treatment; (2) patients were expectedly unable to tolerate either surgery or TAE treatment. Written informed consent was obtained from all patients and a family member before any of the treatment procedures. One of the four therapeutic options was undertaken after initial resuscitation and stabilization. Emergency hepatectomy refers to the performance of surgery with ruptured HCC as the primary treatment, and staged hepatectomy refers to initial hemostasis by TAE (embolization of the feeding artery was performed with gelfoam containing approximately 1 mm^3^ sized absorbable gelatin sponge particles), followed by partial hepatectomy several days later (Hsueh et al. 2012).

The extent of hepatectomy performed depends upon the tumor and residual liver parameters in addition to operating surgeon’s clinical judgment. It was either a lobectomy, a monosegmentectomy, a bisegmentectomy or a wedge hepatectomy with ligation of the feeding artery. Successful hemostasis was defined as hemodynamic stabilization, stable hemoglobin level, and no requirement for further transfusion.

### Statistical analysis

Categorical variables were indicated using counts and percentages (%). The measured data were expressed as mean ± SD. Differences between continuous variables were analyzed using one-way ANOVA and Fisher’s LSD test as post hoc. Categorical variables were compared using χ^2^ test or Fisher’s exact test. Kaplan–Meier survival curves were used for survival analysis, and the difference among groups were compared using log-rank test. Univariate and multivariate Cox-regression were utilized to investigate independent variables which were associated with overall survival. In this study, the final follow-up date was February28, 2015. All patients were followed up for ≥1 year. Statistical analysis was performed using IBM SPSS for Windows version 19 (SPSS, Chicago, IL, USA). Two-tailed *P* < 0.05 was considered statistically significant in all tests.

## Results

### Clinical characteristics of patients

The demographic and clinical data of 162 patients in four groups is summarized in Table 1. There were 136 (84.0 %) men and 26 (16.0 %) women, with a mean age of 58.0 ± 6.4 years. One hundred and thirty-two (81.5 %) patients had hepatitis-B virus (HBV) infection and 142 (87.7 %) had liver cirrhosis. Seventy-four (45.7 %) of the 162 patients were classified as Child-Pugh class A, 44 (27.2 %) as class B, and 44 (27.2 %) as class C. The mean size of lesions was 8.9 ± 1.9 cm. Twenty-five patients (15.4 %) were classified as stage II HCC as proposed by the Liver Cancer Study Group of Japan (Ueno et al. 2002), 56 as stage III (34.6 %), 72 as stage IVA (44.4 %), and nine as stage IVB (5.6 %). The morphology of lesions in all 162 patients included four types: 32 (19.8 %) nodular type, 38 (23.5 %) infiltrative type, 83 (51.2 %) pedunculated type and 9 (5.6 %) cirrhotomimetic type. Among all the characteristics, age, liver cirrhosis, Child Pugh stage, hemoglobin, platelet, international normalized ratio (INR), albumin, total bilirubin, creatinine, modified UICC stage and morphology of lesions were significantly different among four groups (all *P* < 0.05).

**Table 1:** The clinical data of 162 patients with hemorrhagic shock and spontaneous rupture of HCC

Variables	Conservative treatment (n = 35)	TAE alone (n = 21)	Staged surgery (n = 27)	Emergency surgery (n = 79)	Total (n = 162)	P
Age (years)	53.6 ± 5.9	61.5 ± 3.3	58.3 ± 4.6	58.8 ± 6.8	58.0 ± 6.4	<0.001***
Gender (male)	29 (82.9 %)	15 (71.4 %)	24 (88.9 %)	68 (86.1 %)	136 (84.0 %)	0.404
Etiology						0.226
HBV	26 (74.3 %)	18 (85.7 %)	23 (85.2 %)	65 (82.3 %)	132 (81.5 %)	
HCV	3 (8.6 %)	3 (14.3 %)	3 (11.1 %)	6 (7.6 %)	15 (9.3 %)	
Others	6 (17.1 %)	0 (.0 %)	1 (3.7 %)	8 (10.1 %)	15 (9.3 %)	
Liver cirrhosis (yes)	35 (100.0 %)	21 (100.0 %)	20 (74.1 %)	66 (83.5 %)	142 (87.7 %)	<0.001***
Child-Pugh stage						<0.001***
A	3 (8.6 %)	3 (14.3 %)	15 (55.6 %)	53 (67.1 %)	74 (45.7 %)	
B	9 (25.7 %)	9 (42.9 %)	9 (33.3 %)	17 (21.5 %)	44 (27.2 %)	
C	23 (65.7 %)	9 (42.9 %)	3 (11.1 %)	9 (11.4 %)	44 (27.2 %)	
Hemoglobin (g/L)	83.9 ± 6.2	95.7 ± 11.2	91.5 ± 4.6	91.3 ± 9.7	90.3 ± 9.3	<0.001***
Platelet (×10^9^/L)	106.7 ± 14.7	132.1 ± 47.0	117.6 ± 19.9	108.7 ± 16.2	112.8 ± 24.1	<0.001***
INR	2.4 ± 1.3	1.4 ± .2	1.3 ± .2	1.5 ± .3	1.6 ± .7	<0.001***
Albumin (g/L)	29.2 ± 4.8	34.1 ± 3.7	32.6 ± 3.6	33.5 ± 3.4	32.5 ± 4.2	<0.001***
Total bilirubin (mmol/L)	61.9 ± 35.2	20.5 ± 6.3	24.4 ± 8.9	24.9 ± 11.6	32.3 ± 24.3	<0.001***
ALT (IU/L)	182.6 ± 152.6	143.2 ± 59.6	191.8 ± 79.3	148.1 ± 77.2	162.2 ± 98.2	0.094
Creatinine (mg/dL)	1.1 ± .1	1.3 ± .1	1.3 ± .2	1.3 ± .2	1.3 ± .2	<0.001***
AFP (µg/L)	7532.9 ± 5515.6	9136.7 ± 2421.3	9836.3 ± 6619.4	8518.0 ± 6895.8	8605.1 ± 6149.1	0.513
Size of lesions (cm)	8.8 ± 1.6	9.0 ± 2.0	9.0 ± 1.4	8.8 ± 2.3	8.9 ± 1.9	0.959
Multiple tumors (yes)	20 (57.1 %)	9 (42.9 %)	12 (44.4 %)	34 (43.0 %)	75 (46.3 %)	0.546
Portal vein (yes)	12 (34.3 %)	9 (42.9 %)	9 (33.3 %)	29 (36.7 %)	59 (36.4 %)	0.906
Modified UICC stage						0.002**
2	6 (17.1 %)	3 (14.3 %)	1 (3.7 %)	15 (19.0 %)	25 (15.4 %)	
3	6 (17.1 %)	9 (42.9 %)	17 (63.0 %)	24 (30.4 %)	56 (34.6 %)	
4A	17 (48.6 %)	9 (42.9 %)	9 (33.3 %)	37 (46.8 %)	72 (44.4 %)	
4B	6 (17.1 %)	0 (.0 %)	0 (.0 %)	3 (3.8 %)	9 (5.6 %)	
Morphology of lesions						<0.001***
Nodular	1 (2.9 %)	3 (14.3 %)	13 (48.1 %)	15 (19.0 %)	32 (19.8 %)	
Infiltrative	10 (28.6 %)	5 (23.8 %)	0 (.0 %)	23 (29.1 %)	38 (23.5 %)	
Pedunculated	18 (51.4 %)	11 (52.4 %)	14 (51.9 %)	40 (50.6 %)	83 (51.2 %)	
Cirrhotomimetic	6 (17.1 %)	2 (9.5 %)	0 (.0 %)	1 (1.3 %)	9 (5.6 %)	

*AFP* α-fetoprotein, *ALT* alanine aminotransferase, *HBV* hepatitis B virus, *HCV* hepatitis C virus, *INR* international normalized ratio, *UICC* Union for International Cancer Control

** *P* < 0.01; *** *P* < 0.001

### Treatment and outcomes

All patients received initial intravenous fluid resuscitation, supportive care and blood products as required in the intensive care unit immediately after admission. Treatment decisions were taken on individual case basis taking into consideration the patients’ performance status, organ functions, comorbidities and the wishes of the patient and families. Of the 162 patients, 35 (21.6 %) patients received only medical treatment and fluid therapy (conservative treatment group, n = 35) in view of their poor liver function and/or multiple lesions or the wishes of the families. TAE hemostasis was performed in 48 (29.6 %) patients (TAE alone group, n = 21 and staged surgery group, n = 27) in view of their poor liver function, absence of contraindications to the procedure or the wishes of the families. All the 48 patients underwent TAE were of successful embolization. Partial hepatectomy was the definitive treatment in 106 (65.4 %) patients. With respect to the surgical methods used, wedge resection of the tumor was performed in 16 patients, monosegmentectomy in 19, bisegmentectomy in 22, and lobectomy in 49. Seventy-nine patients had surgery immediately after primary resuscitation (emergency surgery group, n = 79), and the remaining 27 underwent staged partial hepatectomy 1–10 d after TAE (staged surgery group, n = 27).

### Causes of death within 30 days

Forty seven of the 162 patients died within 30 days in the hospital. Most of the patients in the conservative treatment group died within 30 days of hospitalization during the first admission, and the chief cause of death in this group was re-bleeding of HCC (21 cases; 65.6 %). In the TAE-only group, 14 patients (66.7 %) were discharged from hospital and 7 died within 30 days. The main cause of death in the TAE-only group was liver failure (4 patients, 57.1 %), and only two (28.6 %) died of re-rupture or re-bleeding of HCC. In the hepatectomy group (emergency and staged), fewer patients died within 30 days (Table 2). The distribution of cause of death did not significantly differ in different groups (*P* = 0.211).

**Table 2:** Causes of death within 30 days

Treatment option	No. of patients	No. of patients who died	Re-rupture or re-bleeding	Hepatic failure	Others	P
Conservative treatment	35	32	21	9	2	0.211
TAE alone	21	7	2	4	1	
Emergency surgery	79	6	4	2	0	
Staged surgery	27	2	0	2	0	
Summary	162	47	27	17	3	

*TAE* transcatheter arterial embolization

### Overall survival

**Fig. 1:**
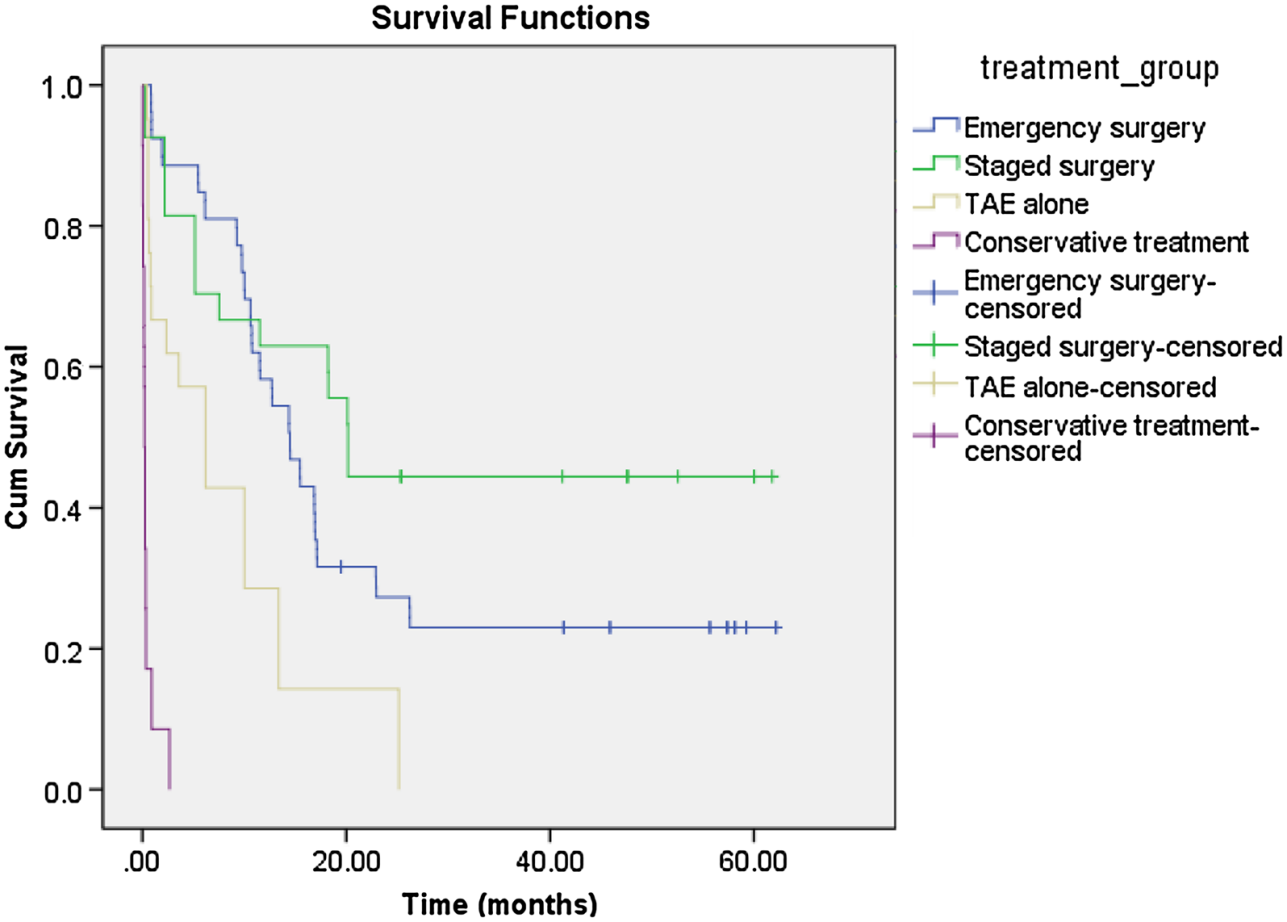
Cumulative survival rates in four treatment groups

**Fig. 2:**
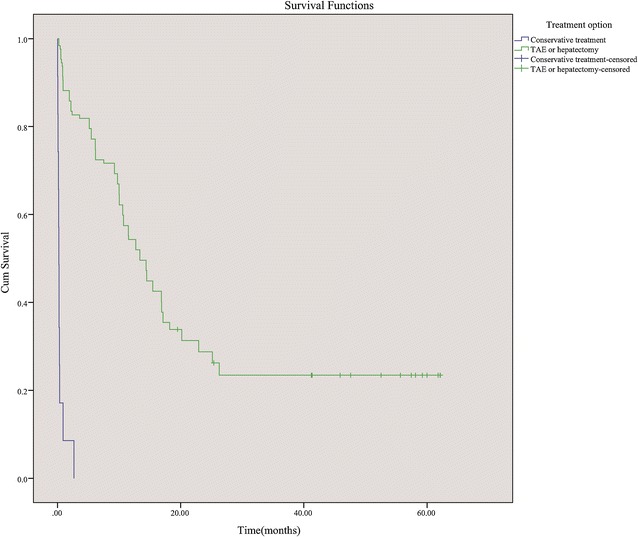
Cumulative survival rates in patients who underwent conservative treatment and TAE/hepatectomy

**Fig. 3:**
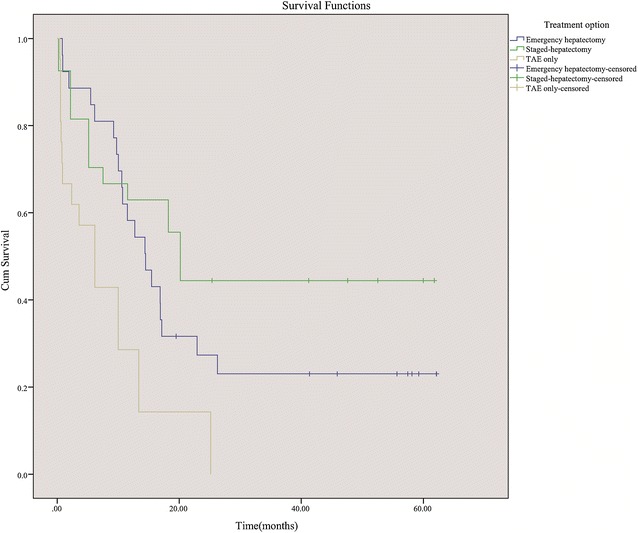
Cumulative survival rates in patients who underwent emergency hepatectomy, staged hepatectomy, or TAE

The survival rates of all 162 patients at 30 days and 1 year were 71.0 % (115/162) and 42.6 % (69/162). The survival rates at 30 days and 1 year in the patients who underwent conservative treatment were 8.6 % (3/35) and 0 % (0/35), respectively. These rates were significantly lower than those receiving either hepatectomy or TAE (88.2 %; 112/127 and 54.3 %; 69/127; χ^2^ = 84.45, *P* < 0.001 and χ^2^ = 33.12, *P* < 0.001). The overall cumulative survival rates among four treatment groups reached significant difference (Log-rank test, *P* < 0.001, Fig. 1). The cumulative survival rates of the patients undergoing either hepatectomy or TAE were higher than those receiving conservative treatment (Log-rank test, *P* < 0.001, Fig. 2). The patients who underwent hepatectomy (emergency or staged) showed better cumulative survival than those receiving TAE alone (Log-rank test, *P* < 0.001; Fig. 3). The survival rate at 30 days for the patients who underwent hepatectomy was 92.5 % (98/106), which was higher than those receiving TAE alone (66.7 %; 14/21; *P* = 0.003). The survival rate at 1 year was also superior in patients who underwent hepatectomy (59.4 %; 63/106) compared to those receiving TAE alone (28.6 %; 6/21) (χ^2^ = 6.73, *P* = 0.009). There was no significant difference between the cumulative survival rates of emergency hepatectomy and the rates of staged hepatectomy (χ^2^ = 2.63, *P* = 0.105).

### Independent variables associated with overall survival

To adjust for potential confounders including all the demographic and clinical characteristics, Cox-regression was utilized to analyze prognostic factors for overall survival. The results of univariate Cox-regression (estimated hazard ratio [HR]) and multivariate model (adjusted HR) adjusted for all other factors were shown in Table 3. Variables significant in both univariate and multivariate regression would be regarded as a prognostic factor for overall survival of patients. Consistent with the result of cumulative survival analysis, stated surgery [hazard ratio (HR) 0.02; 95 % confidence interval (CI) 0.00–0.09; *P* < 0.001], emergency surgery (HR 0.13; 95 % CI 0.03–0.50; *P* = 0.003) and TAE therapy (HR 0.05; 95 % CI 0.02–0.170; *P* < 0.001) were significant protective factors for survival as compared with conservative treatment. Other significant associated factors included etiology, liver cirrhosis, Child-Pugh stage, hemoglobin, AFP, and modified UICC stage.

**Table 3:** Cox-regression of independent variables to overall survival

Variables	Estimated HR (95 % CI)	P	Adjusted HR (95 % CI)	P
Treatment		<0.001***		<0.001***
Conservative treatment	1	–	1	–
TAE alone	0.04 (0.02–0.08)	<0.001***	0.05 (0.02–0.17)	<0.001***
Staged surgery	0.03 (0.01–0.06)	<0.001***	0.02 (0.00–0.09)	<0.001***
Emergency surgery	0.10 (0.05–0.20)	<0.001***	0.13 (0.03–0.50)	0.003**
Age (years)	1.04 (1.01–1.07)	0.019*	0.98 (0.93–1.04)	0.595
Gender (male)	1.24 (0.79–1.95)	0.353	0.37 (0.17–0.81)	0.012*
Etiology		0.028*		<0.001*
HBV	1	–	1	–
HCV	1.30 (0.75–2.23)	0.349	0.97 (0.43–2.19)	0.936
Others	0.36 (0.16–0.82)	0.015*	0.07 (0.02–0.27)	<0.001***
Liver cirrhosis (yes)	7.25 (3.17–16.58)	<0.001***	4.73 (1.76–12.73)	0.002**
Child-Pugh stage		<0.001***		<0.001***
A	1	–	1	–
B	3.52 (2.28–5.44)	<0.001***	5.75 (2.86–11.54)	<0.001***
C	15.54 (9.64–25.05)	<0.001***	16.09 (5.43–47.65)	<0.001***
Hemoglobin (g/L)	0.96 (0.94–0.98)	<0.001***	0.92 (0.87–0.98)	0.006**
Platelet (×10^9^/L)	1.00 (0.99–1.00)	0.422	0.99 (0.97–1.00)	0.057
INR	4.13 (2.97–5.73)	<0.001***	1.51 (0.87–2.63)	0.144
Albumin (g/L)	0.88 (0.84–0.92)	<0.001***	1.03 (0.94–1.13)	0.528
Total bilirubin (mmol/L)	1.07 (1.06–1.09)	<0.001***	1.02 (0.99–1.05)	0.132
ALT (IU/L)	1.00 (1.00–1.01)	<0.001***	1.00 (1.00–1.00)	0.381
Creatinine (mg/dL)	0.48 (0.20–1.14)	0.097	0.15 (0.02–0.99)	0.049*
AFP (µg/L)	1.00 (1.00–1.00)	0.019*	1.00 (1.00–1.00)	0.012*
Size of lesions (cm)	0.98 (0.91–1.06)	0.662	0.80 (0.68–0.93)	0.004**
Multiple tumors (yes)	1.34 (0.95–1.89)	0.094	0.25 (0.09–0.73)	0.011*
Portal vein (yes)	1.13 (0.80–1.61)	0.486	8.63 (2.79–26.66)	<0.001***
Modified UICC stage		0.002**		0.008**
2	1	–	1	–
3	1.13 (0.65–1.97)	0.657	5.20 (1.89–14.32)	0.001**
4A	1.56 (0.92–2.64)	0.098	2.10 (0.75–5.86)	0.157
4B	4.38 (1.92–10.02)	<0.001***	2.98 (0.65–13.55)	0.159
Morphology of lesions		<0.001***		0.247
Nodular	1	–	1	–
Infiltrative	3.29 (1.85–5.83)	<0.001***	1.19 (0.61–2.34)	0.604
Pedunculated	2.14 (1.27–3.62)	0.004**	0.87 (0.48–1.58)	0.657
Cirrhotomimetic	16.79 (7.02–40.18)	<0.001***	1.87 (0.65–5.38)	0.246

*AFP* α-fetoprotein, *ALT* alanine aminotransferase, *HBV* hepatitis B virus, *HCV* hepatitis C virus, *INR* international normalized ratio, *UICC* Union for International Cancer Control, *HR* hazard ratio, *CI* confidence interval

* *P* < 0.05; ** *P* < 0.01; *** *P* < 0.001

## Discussion

This study shows that the incidence of ruptured HCC is more in males, which corresponds with the skewed incidence of HCC towards males observed worldwide. Infective causes of HCC predominate in our patients, which corresponds with the East Asian scenario. Child-Pugh class A predominated in our study, which is not the case in few of the recent publications where class B and C predominate (Jin et al. 2013). This could be because of early referrals in our hospitals.

Some studies have identified that hemorrhagic shock has a negative impact on short-term survival (Kirikoshi et al. 2009; Hsueh et al. 2012; Kim et al. 2012). The degree of acute liver injury, hemoperitoneum and hemodynamic instability had a major impact on 30 day mortality Massive hemoperitoneum of ruptured HCC often leads to hemorrhagic shock, and fluid resuscitation may stabilize these patients. After emergency fluid resuscitation, patients are classified as hemodynamically stable or not, and only some may be suitable for TAE or surgery. While some authors suggest that TAE and hepatectomy are important factors influencing short-term survival (Kirikoshi et al. 2009; Lin et al. 2014; Hsueh et al. 2012; Jin et al. 2013), others regard TAE and staged hepatectomy as prognostic factors (Tan et al. 2006; Hsueh et al. 2012; Kung et al. 2008).

Most HCC tumors have a rich blood supply, and receive most blood from the hepatic artery. Therefore, since the pressure of arterial bleeding is higher, without surgery or TAE, the risk of continuous or repeated bleeding is high with conservative treatment, and impaired liver function can be exacerbated in patients not receiving interventional hemostatic treatment. Chen et al. (2005) suggested that conservative treatment is preferable in patients with ruptured HCC, stating that emergency TAE does not offer significant advantages, as indicated by the disappointing prognosis. However, the present study findings demonstrated that short-term and cumulative survival rates were significantly higher in patients who underwent TAE or hepatectomy than in those who received conservative treatment alone. Multivariate Cox-regression analysis also showed that hepatectomy and TAE therapy were protective factors for survival as compared to conservative treatment. Hsueh et al. (2012) showed that ruptured HCC after TAE has better prognosis than after conservative treatment, and TAE may achieve successful hemostasis for ruptured HCC in 99 % of patients. TAE has replaced other surgical hemostatic methods, such as hepatic artery ligation (Kung et al. 2008; Li et al. 2007; Castells et al. 2001). Although TAE can offer excellent hemostasis in ruptured HCC, we observed in this study that the therapeutic effect is apparently inferior to hepatectomy. Though the surgery may be difficult initially due to hemodynamic instability, staged TAE followed by surgery in select patients can solve this problem as suggested by Li et al. (2009). We opine that the role of TAE as the definitive or preliminary treatment might depend on the post-TAE recovery of liver function and re-evaluation of the patient’s clinical condition, and therefore, treatment should be tailored according to individual requirement. In the present study, post-TAE liver failure was observed in four (19.0 %) patients in the TAE group, which suggests that TAE should be selectively administered in patients with good reserved hepatic function and tolerable coagulopathy.

In this study, 65.4 % patients underwent partial hepatectomy (emergency or staged), 29.6 % underwent TAE and only 21.6 % opted for conservative treatment. This could be because majority of patients were well preserved and fit for surgery in contrast to more numbers of unfit patients in other studies (Jin et al. 2013).

For all the patients in the present study, the 30-day and 1-year survival rates were 71.0 and 42.6 %, respectively, which is reassuring. The 30-day and 1-year survival rates for patients receiving hepatectomy were 92.5 and 59.4 %, respectively, which are higher than those reported in other studies (Liu et al. 2001; Chen et al. 2005; Kirikoshi et al. 2009; Lai and Lau 2006; Yeh et al. 2002; Tarantino et al. 2011; Miyoshi et al. 2010). This could be because of a selection bias due to early referral (72.9 % being Child-Pugh class A or B), better initial resuscitation and preserved liver function. With partial hepatectomy, there is still a hope of cure for select patients even with spontaneously ruptured HCCs. Emergency hepatectomy combines the benefit of hemostasis and a definitive treatment in a single surgical procedure. Some experts suggest that delayed hepatectomy after initial hemostasis might compromise the resection rate, because the rupture can aid in spread of the tumor. Findings from a single-center study concluded that one-stage hepatectomy is a better option for patients with preserved liver function, whereas TAE is a better option for those with poorly preserved liver function (Yang et al. 2014). Many report that TAE is a better modality for hemostasis in HCC rupture (Darnis et al. 2014; Kirikoshi et al. 2009; Lee et al. 2014; Lin et al. 2014; Hsueh et al. 2012; Kim et al. 2012; Jin et al. 2013; Yang et al. 2014), and in select patients, staged hepatectomy may be recommended thereafter (Lai and Lau 2006; Lee et al. 2014; Lin et al. 2014; Hsueh et al. 2012; Tarantino et al. 2011). Thus, for patients arriving in the hospital with ruptured HCC with stable hemodynamics, acceptable liver function, and tumor characteristics indicating resectability, emergency hepatectomy should be considered. For patients with equivocal clinical and laboratory parameters for hepatectomy or questionable expected post-hepatectomy liver function, TAE should be used as a temporary/bridging measure for hemostasis. The strategy of dealing with such difficult cases should be more aggressive to pursue longer survival times. In the present study, 106 patients underwent emergency hepatectomy or staged hepatectomy after TAE, and the 30-day and 1-year survival rates were better than in patients receiving conservative treatment. Patients with ruptured HCC without a prior diagnosis of liver cancer usually have reserve liver function, and can be stabilized with proper fluid resuscitation, thus, making them suitable candidates for surgery.

The limitations to this study are: firstly, the study was of retrospective design. Many patients were transferred from other regional hospitals, of whom, some may have undergone vigorous fluid resuscitation and been stabilized, whereas others may not have been transferred at all; and secondly, there is a possibility of selection bias with only relatively healthy patients being transferred to these referral institutes. We do not consider this population of patients to be representative of the general population of HCC, but the treatment modalities need to be seriously considered in select patients. Thirdly, the treatment choice in this study was based on patients’ general condition. Several demographic and clinical characteristics, such as age, liver cirrhosis, Child-Pugh stage, hemoglobin, platelet, INR, albumin, total bilirubin, creatinine, modified UICC stage, morphology of lesions, were significantly different among four groups. Therefore, a multivariable Cox model was used to adjust for these potential confounders. However, multivariable Cox-regression analysis also showed that liver cirrhosis, Child-Pugh stage and modified UICC stage were associated factors for overall survival. Hence, correlation between treatment adopted and survival is biased by the selection of patients.

In conclusion, by the time patients with ruptured HCC were brought to the emergency department, they were already in hemorrhagic shock. TAE and partial hepatectomy were found to be useful treatment strategies for hemorrhagic shock patients with spontaneous rupture of HCC. Partial hepatectomy, especially staged partial hepatectomy after TAE, tended to provide better survival than TAE alone. A multidisciplinary team comprising of surgeons, oncologists and radiologists should collaborate to implement a definitive treatment. Conservative treatment may be best reserved for patients who are poor candidates for TAE or hepatectomy.

## Abbreviations

HCC: hepatocellular carcinoma
CT: computed tomography
TAE: transcatheter arterial embolization
